# Bionic Microbubble Neutrophil Composite for Inflammation-Responsive Atherosclerotic Vulnerable Plaque Pluripotent Intervention

**DOI:** 10.34133/2022/9830627

**Published:** 2022-06-03

**Authors:** Fangfang Liu, Yang Mao, Jiaqi Yan, Yu Sun, Zhihua Xie, Fei Li, Fei Yan, Hongbo Zhang, Pengfei Zhang

**Affiliations:** ^1^The Key Laboratory of Cardiovascular Remodeling and Function Research, Chinese Ministry of Education, Chinese National Health Commission and Chinese Academy of Medical Sciences, The State and Shandong Province Joint Key Laboratory of Translational Cardiovascular Medicine, Department of Cardiology, Qilu Hospital, Cheeloo College of Medicine, Shandong University, Jinan 250012, China; ^2^Turku Bioscience Centre, University of Turku and Åbo Akademi University, And Pharmaceutical Sciences Laboratory, Åbo Akademi University, Turku 20520, Finland; ^3^Department of Ultrasound in Medicine, The Second Affiliated Hospital of Zhejiang University School of Medicine, No. 88 Jiefang Road, Shangcheng District, Hangzhou 310009, China; ^4^Shenzhen Institute of Advanced Technology, Chinese Academy of Science, Shenzhen 518055, China

## Abstract

Rupture or erosion of inflammatory atherosclerotic vulnerable plaque is essential to acute coronary events, while the target intervene of vulnerable plaque is very challenging, due to the relatively small volume, high hemodynamic shear stress, and multifactorial nature of the lesion foci. Herein, we utilize the biological functionality of neutrophil and the versatility of microbubble in the acoustic field, to form Neu-balloon through CD11b antibody binding. The Neu-balloon inherits the advantage of neutrophils on firming the endothelium adhesion even at shear stress up to 16 dyne/cm^2^ and also maintains the acoustic enhancement property from the microbubble, to accumulate at atherosclerotic lesions under acoustic in an atherosclerotic Apo E^−/−^ mice model. Interestingly, Neo-balloon also has high and broad drug loading capacity, which enables the delivery of indocyanine green and miR-126a-5p into vulnerable plagues *in vivo*. Overall, the bionic Neu-balloon holds great potential to boost on-demand drug transportation into plaques *in vivo*.

## 1. Introduction

Atherosclerotic plaque rupture followed by the thrombosis is the primary cause of acute cardio- and cerebral vascular events with high mortality and morbidity [1–3]. These rupture prone plaques, termed vulnerable plaques, are typically induced by active inflammation hallmarked by macrophage infiltration, and characterized with large lipid core and thin fibrous cap. The atherosclerotic plaques are very difficult to be treated, with a major challenge of targeted drug delivery, since the atherosclerotic plaques are intima-involved lesions in large to medial size artery with high blood flow speed and high shear stress. These hemodynamic characteristics hinder the attachment of conventional drug carriers to the arterial endothelia and their accumulation into the plaque. The targeted motifs or ligands, such as connect selections, integrin, and cell adhesion molecules (CAMs), lend support in strengthening the binding; however, they show low efficiency in animal studies, due to the insufficient affinity to counteract the high shear stress [4]. In the other hand, various cytokines are involved in the inflammation within atherosclerotic vulnerable plaques, and it is difficult to find a universal molecular marker as target for different patients. Moreover, single type of drug is often not sufficient for the treatment of the plaque.

Benefit by the intrinsic homing and drug-carrying property of some living cells, cell-based delivery system has recently shown promising to serve as “decoys” to neutralize proinflammatory cytokines and endotoxins [5–8]. Neutrophils are the largest population of circulating leukocytes. The neutrophils are evidenced to traffic to the injured endothelia, caused by atherogenic factors or inflammatory chemotactic, and they are also found to colocalize with macrophage within vulnerable plaques. After the short life span (one to two days) [1, 9], apoptotic neutrophils are engulfed by macrophage, and this rapid phagocytosis polarizes macrophages to antiatherosclerotic phenotype to restore homeostasis [10]. It has been recently revealed that neutrophil holds the ability to bind to inflammatory endothelium at 4 to 16 dyne/cm^2^ shear stress by cell-flattening, catch bonds, long tethers and so-called “slings” around rolling cells [11, 12]. They can also travel through blood flow and migrate into atherosclerotic lesions [5]. Recently, Wu et al. has applied neutrophil to deliver doxorubicin for brain tumor homing and therapy [13]. Taken together, neutrophil is promising to be an attractive cell-based drug delivery system targeting vulnerable plaques. However, many disadvantages are negligible in terms of this purpose. In patients, the neutrophil is scarcely detected in vulnerable plaques, which means that the target delivery efficiency to vulnerable plaques will not be high. Moreover, the treatment of vulnerable plaques often involves the delivery of biomolecules, such as nucleoids and proteins, and it is almost impossible to deliver those biomolecules with neutrophil since they will be enzymo- and hydrolyzed, and they can also cause unpredictable side effect like degranulation to neutrophil itself. Besides, as drug delivery system, pure cell systems suffered with low drug loading degree, poor experimental repeatability, and variable drug release profiles in different batches, etc.

Microbubbles have been well accepted as one of the multifunctional carriers since they not only can load drug but also have contrast enhancement and targeting augment properties under ultrasound. Ultrasound gives not only the real-time tracing of the microbubbles but facilitates the enrichment of microbubbles at the target by several biophysical mechanism [14], including acoustic radiation force [15] and ultrasound-targeted microbubble destruction (UTMD) effect [16]. Drugs can be delivered to atherosclerotic plaques region via microbubbles under ultrasound [17]. However, the microbubbles themselves are not perfect delivery system for vulnerable plaques. First, the rheological behavior of microbubbles is similar to that of red blood cells, which tends to keep them close to the axial center of the bloodstream [18]. Second, microbubbles' floatation under high shear stress limits their adhesion to rupture-prone site of plaques. Even antibody-conjugated microbubbles can be easily dissociated from the atherosclerotic endothelia. Moreover, the microbubbles also suffer with low drug loading capacity since it only has a thin liposome layer for retaining the drugs.

**Scheme 1:**
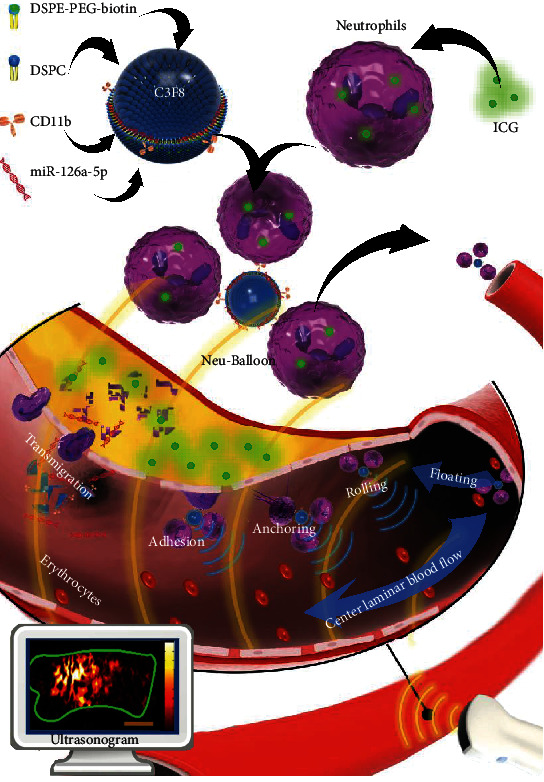
Neu-balloon preparation and in vivo function introduction.

In this study, to manipulate the targeted intervention of atherosclerotic vulnerable plaque, we constructed neutrophil microbubble composite, coincided as Neu-balloon (Scheme 1). MiR-126a-5p has been reported to be involved in the differentiation of immunocytes, angiotensin II antagonization, and lipid metabolism which is beneficial to atherosclerotic plaque stabilization and regression [19–21]. We entrapped the indocyanine green into neutrophils as the example of hydrophilic small molecules while attached miR-126a-5p on the microbubble electrostatically as the example of biomolecules. The Neu-balloons could be traced ultrasonically to the vulnerable plaque site with the acoustic enhancement from the microbubbles and firmly attached and delivered the drugs into vulnerable plaques by the neutrophils. In a word, the Neu-balloons can rapidly target and firmly attach to vulnerable plaques, to efficiently codeliver both enzyme stable small molecules and sensitive biomolecules for advanced vulnerable plaques intervention.

## 2. Results and Discussion

### 2.1. Preparation and Characterization of Neu-Balloons

**Figure 1:**
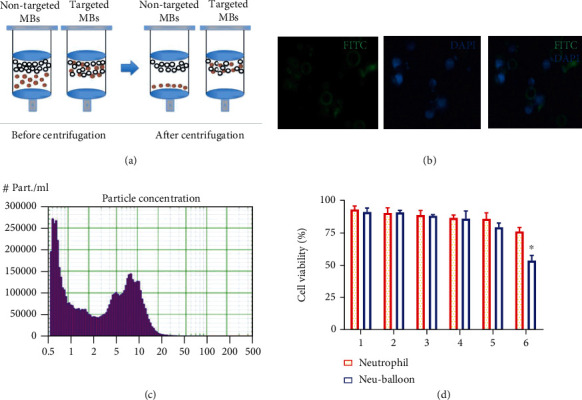
Neu-balloon preparation and characterization. (a) Isolation of unconjugated neutrophils from assembled Neu-balloons. (b) Formation of the Neu-balloons consisting of FITC-labeled microbubbles and conjugated neutrophils (with nuclei stained by DAPI), as imaged by fluorescent microscopy (scale bar = 20 *μ*m). The resulting format was 3.4 ± 1.2 neutrophils per microbubble. (c) The size distribution curve of Neu-balloon with an obvious peak of 8.4 ± 1.2 *μ*m. (d) Evaluation of the neutrophils viability after conjugated to MB_CD11b_ from 1 h to 6 h after preparation (^∗^*P* < 0.05 vs. neutrophil).

The synthetic process of Neu-balloon consisted four steps. First, neutrophils with an average cell size of 6.7 ± 0.5 *μ*m (Figure S1A) were highly purified and isolated via a Percoll gradient separation method, to maintain the max biobinding activity [6]. Second, microbubbles (the average size 1.6 ± 0.6 *μ*m, Figure S1B) conjugated with CD11b antibodies (MB_CD11b_) were prepared via biotin-avidin conjugation. Third, the Neu-balloon was assembled by mixing the cells and MB_CD11b_ together at the ratio of 1 : 100. Last, unconjugated neutrophils and microbubbles were separated by gradient centrifugation (Figure 1(a)). The resulting format was 3.4 ± 1.2 neutrophils per microbubble (Figure 1(b)), and the typical size was 8.4 ± 1.2 *μ*m (Figure 1(c)). Although neutrophils are short-lived cells, this synthetic procedure is mild and fast, and did not affect their viability (Figure 1(d)). We also prepared IgG (MB_IgG_) and anti-CD11b antibody (MB_CD11b_) conjugated microbubbles by biotin-avidin method as controls.

### 2.2. In Vitro Acoustic Enhancement and Stability of Neu-Balloons under UTMD

**Figure 2:**
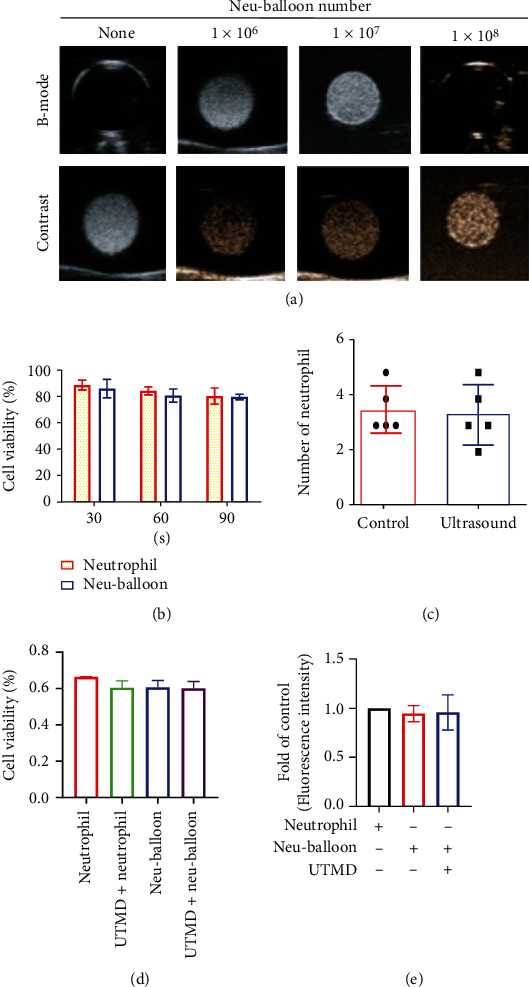
Acoustic enhancement could be observed at B-mode and contrast imaging mode. (a) Take into account the good signal-to-noise ratio of contrast imaging mode, good enhancing effect was obvious at the concentration higher than 1 × 10^7^/ml. (b) Exposure to ultrasound (500 Hz, 5%, 0.35 MPa, 255 W/cm^2^) for 90 seconds did not decrease the cells' viability. (c) Exposure to the same acoustic condition did not affect the formation of Neu-balloons either. The number of neutrophils connected per microbubble was 3.4 in average in both groups. (d) When the acoustic energy was increased to higher level for UTMD, the burst of microbubble-part did not affect the viability of the neutrophil-part of the Neu-balloons. (e) The UTMD would not affect the inflammation-responsive effect of the neutrophil-part as the ICG fluorescent signals from the neutrophils adhered onto the ox-LDL-stimulated MAECs differed nonsignificantly with or without UTMD.

Acoustic enhancement could be observed at minimal concentration of 1 × 10^7^/ml by Vevo 2100 (VisualSonics, Toronto, Canada) with a MS250 nonlinear transducer (center frequency 18 MHz) (Figure 2(a)). Exposure to ultrasound (500 Hz, 5%, 0.35 MPa, 255 W/cm^2^) for 90 seconds did not decrease the cells' viability or formation of Neu-balloons (Figures 2(b) and 2(c)), which is similar to the results of Chen et al. [22]. Since higher energy is required for UTMD, we tested the bioactivity of the residual neutrophils after burst of the microbubble-part of the Neu-balloons, by testing their viability and adhesive capability to inflamed endothelium. Indexed with the endocytosed ICG (details in Section 2.4), the adhesion of neutrophils or Neu-balloons (equivalent at the number of neutrophils at 10^8^/ml) to ox-LDL stimulated MAEC was examined with fluorescent readings. As shown in Figures 2(d) and 2(e), the UTMD effect (output 1.6 W at frequency of 1 MHz, duty cycle 50%, duration 5 minutes) did not affect the activity of the neutrophil-part.

### 2.3. Bionic Targeting Performance against High Shear Stress

**Figure 3:**
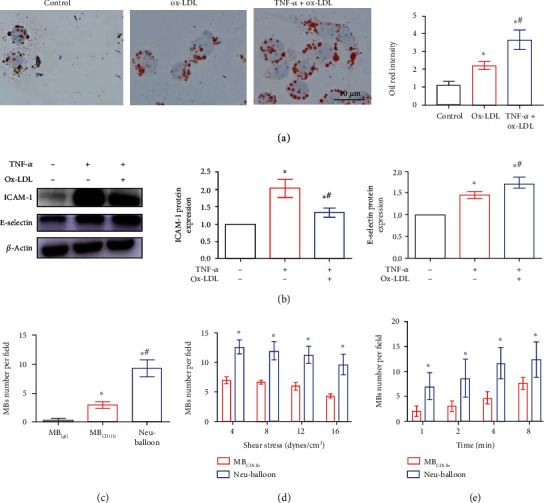
Bionic targeting performance against high shear stress *in vitro*. (a) The inflammatory cell model was developed by MAEC stimulated with TNF-*α* and ox-LDL. More lipids were detected in this model indicating active phagocytosis of lipoprotein, as evidenced by Oil-red staining with the intensity normalized by the cell counts (scale bar = 10 *μ*m). (b) As response to inflammatory stimuli, ICAM-1 and E-selectin were highly expressed as demonstrated by Western blot analysis (^∗^*P* < 0.05 vs. control, ^#^*P* < 0.05 vs. stimulating with only TNF-*α*). (c) Good static affinity of Neu-balloons to inflammatory MAEC was demonstrated compared to MB_IgG_ and MB_CD11b_ (^∗^*P* < 0.05 vs. MB_IgG_, ^#^*P* < 0.05 vs. MB_CD11b_). (d) The high affinity of Neu-balloon to the inflamed MAEC could be maintained at high shear stress condition, even at shear stress as higher as 16 dynes/cm^2^ (^∗^*P* < 0.05 vs. MB_CD11b_) created in a flow chamber mimicking the shear stress status in a medium- or large-sized artery. (e) The ability of Neu-balloon to counter high shear stress could be dominant with prolonged circulating time (^∗^*P* < 0.05 vs. MB_CD11b_).

What encountered in real-life clinical challenge on atherosclerotic vulnerable plaque drug delivery is that the hemodynamic circumstance is very different from that in tumours [23]. The high blood flow speed and high shear stress exists in medial- to large-calibre arteries, which is prevalent sites for atherosclerotic plaques. To validate the inflammation-responsiveness capability of the Neu-balloon against high shear stress circumstance, mouse aortic endothelial cells (MAEC) were stimulated by TNF-*α* (10 ng/ml) + oxLDL (60 mg/l) to develop the inflammatory cell model (Figure 3(a)), which mimics the atherogenic environment of inflammation and high cholesterol. As a result, the expression of intercellular cell adhesion molecule 1 (ICAM-1) and E-selectin, which are abundant on the activated vascular endothelium membrane accounting for the inflammation responsive adhesion of neutrophils, had significantly increased (Figure 3(b)). As seen in Figure 3(c), Neu-balloon had strongest binding affinity to stimulated MAEC among MB_IgG_ and MB_CD11b_ (9.33 ± 2.51 Neu-balloon per cell vs. 0.33 ± 0.57 MB_IgG_ per cell vs. 3.00 ± 1.00 MB_CD11b_ per cell, respectively, *P* < 0.05).

To further test the shear-resistant endothelial adhesion of Neu-balloon, the parallel plate flow chamber was used to create different shear stress flow environment in which the TNF-*α* + ox-LDL-stimulated MAEC were seeded on the bottom. Within the shear stress range of 4 to 16 dyne/cm^2^, Neu-balloon demonstrated significant higher binding affinity than MB_CD11b_ (*P* < 0.05) after circulating for 4 mins (Figure 3(d)). Additionally, keeping the shear stress constant at 4 dyne/cm^2^, the attachment of Neu-balloon was significantly higher than that of MB_CD11b_ over time from 1 minute to 8 minutes (*P* < 0.05 for all, Figure 3(e)). Furthermore, the binding of MB_CD11b_ or Neu-balloon to MAEC was depleted by 2,4-disubstituted thieno [2,3-c] pyridine (A-205804), a selective lead inhibitor of E-selectin and ICAM-1, whenever at static or high shear stress state (Figure S2A, B, C).

Inflammation and response of immune cells are key mechanisms in the development and progression of atherosclerosis [7]. The most abundant white blood cell in the circulation, the neutrophil granulocyte (accounting for up to 75% of the total population of white blood cells), has until recently been reported to play a complex and nonredundant role in atherosclerosis-associated inflammation. This is not only restricted by the activity of the neutrophils themselves but also involves their interaction with immune and nonimmune cells [24]. Neutrophils are the main cell population that interacts with endothelium, suggesting the ongoing recruitment of leukocytes to atherosclerotic lesions [25].

In our study, we isolated neutrophils from peripheral blood of an atherosclerotic Apo E^−/−^ mice mode. Activated by high cholesterol and relevant inflammation, these neutrophils will “target” and subsequently transmigrate into the plaque in response to endothelial injury [26]. Within the plaque, unlike the most activated macrophage, these transmigrated neutrophils sacrifice themselves to control the inflammation and evoke the antiatherosclerotic effect. It assembles the immune system innate to defense invading pathogens [27]. On the other hand, these activated neutrophils were identified by highly expressed CD11b and multiple ligands, especially E-selectin, L-selectin, and VLA-4. These molecules play a core role on recruiting neutrophils to arterial endothelial [7]. Together with pseudopodium and active deformation, Neu-balloon was validated in this study capable to resist the characteristic high shear stress in medium- or large-sized arteries that are the predilection sites of atherosclerotic vulnerable plaques.

### 2.4. Drug Loading to Neu-Balloons

In addition to the bionic adhesion and acoustic tracing, the more important role of the Neu-balloon was to delivery different types of drugs simultaneously at large volume. Drugs could be encapsulated in microbubbles and/or neutrophils according to types. We took ICG as an example of hydrophilic small molecule which is stable against the lysis and acidity within the neutrophil. ICG was loaded into the isolated neutrophils through endocytosis, whilst biomolecules like siRNA [28], and miRNA are not suitable to be loaded directly into neutrophils due to the sensitivity to biodegradation. Since the potentially beneficial effect of miR-126-5p on atherosclerosis, we attached the miR-126a-5p onto the microbubble-part of the Neu-balloon. As shown in Figure S3, the relative fluorescent intensity correlated logarithmically with the concentration of ICG-loaded Neu-balloon (*R*^2^ = 0.97), and the maximal loading capacity of 2.9 *μ*g/10^5^ neutrophils. MiR-126a-5p was loaded onto the microbubbles by electrostatic adsorption with the maximal binding of 2 *μ*g/10^8^ microbubbles. The binding capacity was similar to the reports by Yuan et al. [29] and Liu et al. [30]. As a result, one typical batch of Neu-balloon could carry 2 *μ*g miR-126a-5p and 11.9 mg ICG.

### 2.5. In Vivo Validation of the Multiple Function of Neu-Balloons to Vulnerable Plaques

**Figure 4:**
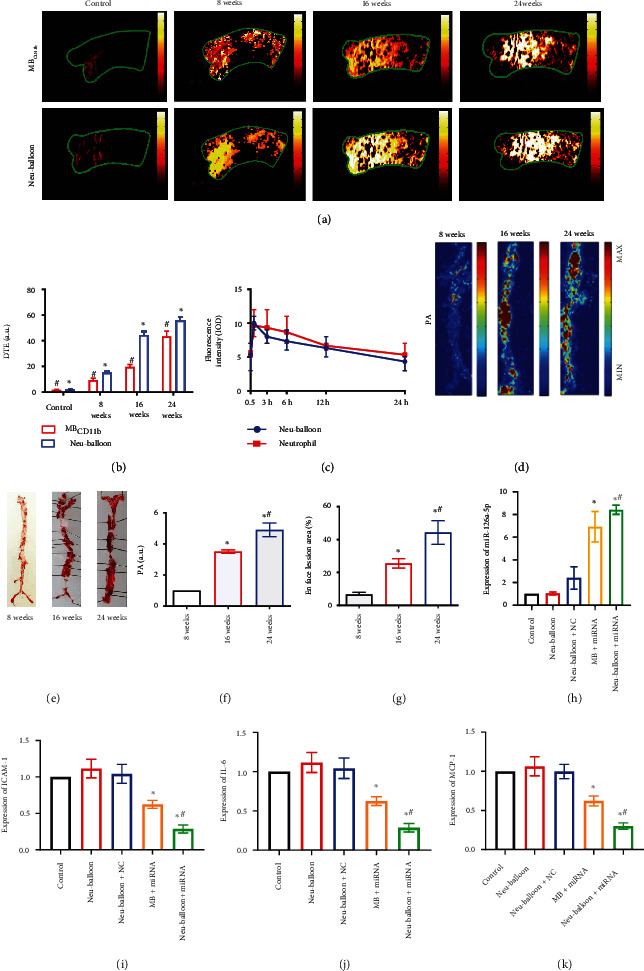
Multiple functionalities *in vivo*. (a) Take advantage of the bionic inflammation-responding effect against high shear stress and the acoustic enhancement capability, Neu-balloons could provide higher image intensity and acoustic contrast compared to MB_CD11b_ on an atherosclerotic vulnerable plaque Apo E^−/−^ mouse model in stages of atherosclerosis. (b) The statistical analysis showed the same results as those in (a). Also, the video intensity at the same imaging window increased significantly along the feeding period (^#^*P* < 0.05 compared to control, ^∗^*P* < 0.05 compared to 8 weeks). (c) With enhanced transportation of Neu-balloons by UTMD, ICG fluorescent intensity within the atherosclerotic plaque transported by Neu-balloon at 5 × 10^8^ neutrophils/ml was similar to neutrophil alone at 1 × 10^9^ neutrophils/ml, as imaged by photoacoustic microscopy. Moreover, this fluorescent signal could last for 24 hours. (d) The photoacoustic signal from the transported ICG. (e) Gross image of the aorta plaques stained by Oil O Red. The fluorescent signal was colocalized with lipid contents within the aorta. (f) Statistical analysis of the fluorescent signals from the aorta. (g) Statistical analysis of the Oil O Red stained histograms. The fluorescent signal was enriched parallel to the severity of the atherosclerotic lesion. (h) In mice fed with atherogenic diet for 24weeks, the expression level of miR-126a-5p within the atherosclerotic plaques was also significantly higher with Neu-balloon injection than that with miR-126a-5p-loaded traditional microbubbles injection. (i) The expression of ICAM-1 decreased significantly by miR-126a-5p transportation. Lowest level was evidenced with mir-126a-5p delivered by the Neu-balloon. (j) The expression of IL-6 among groups showed similar trend. (k) The expression of MCP-1 decreased also in accordance with the expression of miR-126a-5p within the plaques. PA indicates the fluorescent intensity acquired by photoacoustic microscopy. NC indicates the negative control microRNA. For (f) and (g), ^∗^ indicates *P* < 0.05 compared to Apo E^−/−^ mice fed with atherogenic diets for 8 weeks, whereas ^#^ indicates *P* < 0.05 compared to Apo E^−/−^ mice fed with atherogenic diets for 16 weeks. For (h–k), ^∗^ indicates *P* < 0.01 compared to mice injected saline as controls, whereas ^#^ indicates *P* < 0.01 compared to mice injected miR-126a-5p-loaded microbubbles.

*In vivo* validation of the multiple function of the Neu-balloon was performed on an atherosclerotic Apo E^−/−^ mice model. As demonstrated by Oil O Red and Picric-Sirius red histological staining, as well as anti-*α*-actin and anti-CD68 immuno-histological staining, macrophage and lipid contents within plaques increased over time, whereas the collagen and smooth muscle cells decreased simultaneously. Thus, leading to the augment of plaque vulnerability represented as the increased vulnerable index (Figure S4A, B). In consideration of the animal ethics and possible death from excessive injection, Neu-balloons with ICG and miR-126a-5p duo-loading and MB_CD11b_ were injected into the same mouse via tail vein in sequence at 40 minutes intervals to ensure eliminating the residual effects from the prior composites. As shown in Figures 4(a) and 4(b), Neu-balloon had higher mean video intensities than MB_CD11b_ no matter in mice fed with atherogenic diet for 8, 16, or 24 weeks. Moreover, the longer the atherogenic diet was fed, the more significant acoustic enhancement was observed in the aortic artery (Figure 4(b)). Statistically, the macrophages and vulnerability index both correlated positively with the acoustic intensity acquired from the aortic arches of the mice (*r* = 0.90 and 0.81, respectively, *P* < 0.01 for both, Figure S5A, B). These results indicated that Neu-balloons could be enriched along with inflammation at high shear stress setting within the aortic artery.

The Neu-balloon were destructed by the ultrasound-targeted microbubble destruction (UTMD) effect at the very site with acoustic enhancement through the aortic arch scanning window. The transportation of ICG and miR-126a-5p into plaques were determined by fluorescent imaging and RT-PCR. We tested the fluorescent intensity within the plaques in mice at time 0.5 h, 1 h, 3 h, 6 h, 12 h, and 24 h after tail vein injection. The transportation of ICG via the Neu-balloon was confirmed, with highest peak intensity at 1 hour after injection and half-life of 24 hours (Figure 4(c)). Much stronger fluorescent signal was detected in the advanced plaques (Figures 4(d) and 4(e)), where the signal intensity increased along with the lipid-rich area (*R*^2^ = 0.89, *P* < 0.01, Figures 4(f) and 4(g) and Figure S6). Besides, fluorescent signals were observed within inflammatory plaques in a carrier concentration dependent way.

Moreover, the transportation efficiency was significantly improved by the conjugated microbubbles. In Apo E^−/−^ mice fed with atherogenic diet for 24 weeks, ICG fluorescent intensity within the atherosclerotic plaque transported by Neu-balloon at 6 × 10^8^ neutrophils/ml was similar to neutrophil alone at 1 × 10^9^ neutrophils/ml (Figure 4(c)). As for miR-126a-5p, the expression within the plaques was measured at 48^th^ hours after the injection. At the same concentration of Neu-balloon at 6 × 10^8^ neutrophils/ml, the expression level within aortic plaques was significantly high compared to those injected with miR-126a-5p-loaded microbubbles and negative control microRNA-loaded Neu-balloons (Figure 4(h)). We further interrogated the level of proinflammatory factors within the plaque after injecting the miR-126a-5p-loaded Neu-balloons. The macrophage chemoattractant protein-1 (MCP-1), interleukin-6 (IL-6), and ICAM-1 were significantly lowered by the transported miR-126a-5p. The anti-inflammatory effect was much overt than miR-126a-5p carried by traditional microbubbles, although both were augmented by the UTMD (Figures 4(i)–4(k)). The results were aligned with the expression of miR-126a-5p within the plaques, demonstrating not only the beneficial effect of miR-126a-5p on inflammation alleviation but also the effective bionic targeting to the vulnerable plaque of the Neu-balloon, as well as the versatile drug delivery capability with sufficient biosafety (Figure S7).

The dimeric format of the Neu-balloon could reinforce each other. As for the aspect of targeting, the microbubbles in the acoustic field can drag the neutrophils off the high-speed laminar blood flow center to facilitate the interaction between neutrophils and artery wall. Then, neutrophils tow the microbubble heading for the atherosclerotic plaque strongly in a bionic way. As demonstrated in this study, Neu-balloon could attach to inflamed endothelia more effectively under high shear stress condition (up to 16 dynes/cm^2^) *in vitro* and were able to enhance the vulnerable plaque in an inflammation-responsive manner *in vivo* when compared with MB_CD11b_. As for the aspect of drug delivery, we take advantage of the large intracellular volume of neutrophils and larger surface of the microbubbles to carry different types of drugs. The UTMD used in our study could enhance the delivery of the microRNAs loaded onto the microbubbles, as well as the extravasation of the neutrophil-part of the Neu-balloon into the plaques. The transportation efficacy was significantly higher than traditional antibody-conjugated microbubbles. As for the aspect of tracing, the identification of vulnerable plaques and tracking of the delivery were simultaneously accomplished acoustically with the help of microbubble-part or fluorescently or photoacoustically with the help of fluorescence loaded on the neutrophil-part of the Neu-balloon. These results make the Neu-balloon a promising conveyer for gene therapy like CRISPR technology [31].

## 3. Conclusion

In conclusion, Neu-balloons were successfully developed and applied for accurate noninvasive identification of atherosclerotic vulnerable plaque, and high-efficient drug delivery into inflammatory plaques. The merits of the Neu-balloon shed light on not only noninvasive diagnosis but also on-demand therapy of vulnerable plaques.

## 4. Materials and Methods

### 4.1. Isolation of Peripheral Neutrophils

Percoll gradient method described by Wu et al. was used for isolating neutrophils from the whole blood. [13] Blood samples of male Apo E^−/−^ mice were collected and purified by centrifugation (400 g, 10 minutes, 4°C). The cell pellets were then diluted in PBS containing ethylene diamine tetra acetic acid and carefully added into the top of a three-layer Percoll (GE Healthcare Bio-Sciences AB, Uppsala, Sweden) with gradients of 78%, 69%, and 52%. After centrifuge at 1500×g for 30 min at room temperature, the neutrophils were withdrawn from the 78% layer and the 69%/78% interface. Residual erythrocytes were lysed at 4°C to obtain high-purity neutrophils. The size of the isolated neutrophils was measured by an automated cell counter (Countess 3, Thermo Fisher Scientific, Waltham, MA USA).

### 4.2. Neu-Balloon Assembly

The biotinylated microbubble filled with a perfluoro propane (C3F8) core were made by sonification. The shell of the microbubble was composed of 59.4 mol% 1,2-distearoyl-sn-glycerol-3-phosphocholine (DSPC; Sigma-Aldrich, Netherland), 4.1 mol% 1,2-distearoyl-sn-glycerol-3-phosphoethanolamine (DSPE)-PEG (2000) (Avanti Polar Lipids, USA), 0.8 mol% DSPE-PEG (2000)-biotin (Avanti Polar Lipids, USA), and 0.8 mol% (2,3-dioleoyloxy-propyl)-trimethylammonium (DOTAP; Avanti Polar Lipids, USA). Microbubbles (10^9^/ml) were sealed with C3F8 gas to prevent deflation.

To make microbubbles conjugated, 100 *μ*l biotinylated microbubbles were washed twice with PBS/C3F8 and centrifuged (400 g, 3 min, 4°C). Next, 1 mg/ml streptavidin (Sigma-Aldrich, Netherland) was added, and the mixture was incubated at 24°C for 25 min. Superfluous streptavidin was removed by centrifugation. Next, 1 *μ*g biotinylated rat-anti-mice-CD11b (eBioscience, USA) was added, and the mixture was incubated at 24°C for 25 min. Final concentration of the microbubbles was determined using a Multisizer 3 Coulter Counter (Beckman Coulter, Fullerton, CA, USA). Neutrophils were then incubated with anti-CD11b antibody-conjugated microbubbles in a 100 : 1 ratio under continuous rotation at room temperature for 25 min.

The sizes of the microbubble and the Neu-balloon were measured by dynamic light scattering technique (Zetasizer Nano ZS, Malvern Panalytical, Malvern, UK) The number of microbubbles per Neu-balloon was examined using fluorescent microscopy (Eclipse Ti2-E, Nikon, Japan) with the microbubbles and neutrophils labelled with FITC and DAPI separately.

### 4.3. Indocyanine Green (ICG) and MiR-126a-5p Loading

ICG was dissolved in 100 *μ*l DMSO and mixed with 400 *μ*l DMEM (plus 10% fetal bovine serum) to a final concentration of 2 mg/ml. ICG (300 *μ*l), serum-free DMEM (300 *μ*l), and protamine sulfate solution (5 *μ*l) were mixed to a final concentration of 10 mg/ml. The neutrophils were dissociating evenly with pipette three times. The prepared protamine/ICG solution was added and incubated for 1 hour at 37° C, then centrifuge at 400 rpm for 5 minutes. To load microRNAs onto the microbubbles, 10 *μ*g/ml miR-126a-5p (5′-CAU UAU UAC UUU UGG UAC GCG-3′) was added into the cationic lipid microbubbles and incubated on a flat rocker for 30 minutes. Microbubbles loaded with the miR-126a mimics (5′-UUC UCC GAA CGU GUC ACG UTT-3′) were also prepared as the negative control.

The loading capacity of ICG and miR-126a-5p was calculated separately. After the ICG-loaded neutrophil was prepared, the ICG loading capacity was calculated as the (mass of loaded ICG/total number of neutrophils) through the relationship between the fluorescent intensity and the mass. Similarly, the binding efficacy of miR-126a-5p to microbubbles was determined by the optical density (OD) at 260 nm.

### 4.4. Cell Viability Assay

Cell viability assay involved use of Cell Counting Kit-8. Neutrophils (5 × 10^3^ per well) or Neu-balloon (5 × 10^3^ per well) were seeded in a 96-well plate in 5% CO_2_ at 37°C. After removing the media, cells were incubated with CCK-8 reagent for 2 h. OD was measured at 450 nm. Cell viability was calculated and expressed as [(OD_Neutrophil_ + OD_blank_)/(OD_control_ + OD_blank_)] × 100%.

### 4.5. Inflammation Responsive Adhesion of Neu-Balloon in Static Condition *In Vitro*

The MB_IgG_, MB_CD11b_, or Neu-balloon (5 × 10^6^/ml) was added into a 24-well plate precoated with MAEC (10MU-002, iXCells Biotechnology, USA) stimulated by 10 ng/ml TNF-*α* and 60 mg/l ox-LDL. The plate was sealed, inverted, and rotated for 5 min. PBS was used to remove the free Neu-balloon and MBs, and then, the numbers of attached Neu-balloon or MBs were counted under an optical microscope (Olympus, Tokyo, Japan) at five random bright fields of view. In each microscopic view (40x), 9 equivalent fields were partitioned with 3 columns and 3 lines. The counted numbers of attached Neu-balloon or MBs were expressed as MB numbers/field.

### 4.6. Resistance to High Shear Stress *In Vitro*

The parallel-plate flow chamber setup was reported previously [32]. The flow devices were maintained in 37°C incubator with 5% CO_2_. The Neu-balloon, MB_CD11b_, and MB_IgG_ were drawn into MAEC-coated *μ*-Slide I Luer 0.4 mm length chamber (Lot No. 80176, ibidi, German) at a shear stress of 4 dynes/cm^2^ for 1, 2, 4, and 8 min. Then, these three kinds of microbubbles were exposed to a range of lower shear stress of 4, 8, 12, and 16 dynes/cm^2^ for 4 min. After then, the numbers of attached Neu-balloon or MBs were counted and expressed similarly as those aforementioned.

### 4.7. Western Blot Analysis

Total protein of MAEC was extracted, and protein concentrations were examined by a BCA assay kit. Polyvinylidene fluoride membranes were incubated with specific primary antibodies for ICAM-1, E-selectin, and *β*-actin at 4°C for 12 h and then incubated with horseradish peroxidase-conjugated secondary antibodies. Protein bands were visualized by enhanced chemiluminescence kit. The individual bands' densitometric intensity (area × density) on Western blots was measured by the ImageJ software. Sample loadings were normalized to *β*-actin expressions.

### 4.8. Atherosclerotic Animal Model

All male Apo E^−/−^ mice (10 weeks old) and C57 mice were purchased from the Southern Medical University. The study protocol was approved by the Medical Ethics Committee on Animal Research of the Shenzhen Institutes of Advanced Technology, Chinese Academy of Sciences (Ethics No. KY2016-090). All procedures followed the principles of laboratory animal care and guidelines from the National Institutes of Health. Sixty Apo E^−/−^ mice were assigned into 3 groups and fed with atherogenic diet [0.25% cholesterol (Shanghai Lanji Technology), 15% cocoa butter] for 8 (*n* = 10), 16 (*n* = 10), or 24 (*n* = 40) weeks, respectively. Age-matched C57 mice (*n* = 15) served as the control group were also involved following the same grouping and feeding protocol.

### 4.9. Acoustic Tracing of the Bionic Behavior of Neu-Balloon *In Vivo*

Ultrasonography was performed in 10 animals from each group *in vivo* using Vevo 2100 (VisualSonics, Toronto, Canada) with a MS250 nonlinear transducer (center frequency 18 MHz, lateral resolution 165 *μ*m, axial resolution 75 *μ*m). After anaesthetized by isoflurane in oxygen (2 l/min), 50 *μ*l MB_CD11b_ or Neu-balloon (1 × 10^7^) was injected through the tail vein, followed by 0.1 ml normal saline injection. Four minutes later, 250 ultrasonic frames at the long axis view of the aortic arch were acquired from the right parasternal window as described previously [33].

### 4.10. ICG Transportation *In Vivo*

In anesthetized mice, ICG-loaded neutrophil without MB conjugation or ICG-loaded Neu-balloons were injected through tail vein access, respectively, in 10 mice divided equally. After euthanasia, aortas of mice in each group were dissected for fluorescence assay. The dissected aortas were embedded in ultrasonic coupling agent and imaged at emission wavelength 780 nm by home-made photoacoustic microscopy (spatial resolution 100 *μ*m).

### 4.11. Real-Time PCR

Twenty Apo E^−/−^ mice fed with atherogenic diet for 24 weeks were involved in the level of miR-126a-5p and the proinflammatory factor mRNAs quantification. Three inflammatory factors, ICAM-1, IL-6, and MCP-1 were examined. Total RNA from the aorta was isolated using Trizol reagent (Invitrogen). cDNA was prepared from 1 *μ*g RNA using TaqMan Reverse Transcription Reagents (Applied Biosystems, Carlsbad, CA, USA). Real-time PCR was performed using either Taqman probes (with the TaqMan Gene Expression Master Mix, all from Applied Biosystems) or the SYBR Green method (SsoFas EvaGreen Supermix, from Bio-Rad, Hercules, CA, USA) with primers (forward 5′-CCGGCCATTATTACTTTTGG-3′ and reverse 5′-TATGGTTTTGACGACTGTGTGAT-3′ for mmu-mir-126a-5p; forward 5′-CAGCCAGATGCAGTTAACGC-3′ and reverse 5′-GCCTACTCATTGGGATCATCTTG-3′ for MCP-1; forward 5′-CGCTGTGCTTTGAGAACTGTG-3′ and reverse 5′-ATACACGGTGATGGTAGCGGA-3′ for ICAM-1; forward 5′-AGTCACAGAAGGAGTGGCTAAG-3′ and reverse 5′-GAGGAATGTCCACAAACTGATA-3′ for IL-6). After amplification (Bio-Rad CFX96), the relative mRNA expression level was assessed by the 2 − ΔΔCT method.

### 4.12. Histology and Immunohistochemistry

The right carotid arteries were swiftly removed. Each specimen was fixed with 4% paraformaldehyde fixative and embedded in paraffin for haematoxylin and eosin (HE) staining. For immunostaining, serial cross-sections with a thickness of 5 *μ*m were stained with an anti-SMC antibody (1 : 200 dilution; Abcam, Cambridge Science Park Cambridge, UK) and an anti-CD68 antibody (1 : 200 dilution; Abcam, Cambridge Science Park Cambridge, UK) for characterization of smooth muscle cells and macrophage. Secondary antibodies (Maixin Bio, China) were used according to protocols of horseradish peroxidase and diaminobenzidine chromogenic technique. Gross lipid component of the aorta was evaluated by Oil Red staining. Tissues from the heart, spleen, lung, kidney, and liver were also stained by HE to evaluate the biosafety of the injected Neu-balloon.

### 4.13. Statistical Analysis

All data were analyzed by SPSS 18.0 (Chicago, USA). *P* < 0.05 was considered statistically significant. Continuous variables are presented as the mean ± standard deviation. Differences in multiple groups were analyzed using one-way ANOVA. The significance of the differences between the two groups was tested using Dunnett's *T*3 test. The relationships between acoustic or fluorescent intensity and histological data were analyzed by Pearson correlation analysis.

## Data Availability

All data needed to evaluate the conclusions in the paper are presented in the paper and supplementary materials. And additional data are available from the corresponding author upon reasonable request.

## Supplementary Materials

Supplementary Materials The supplementary materials are available by the publisher as online-only content, linked to the online manuscript. All the supplementary materials are summarized in a single file, including text, figures, and tables, as listed below. Figure S1: size distribution curve of the isolated neutrophils and prepared microbubbles. Figure S2: the inflammatory cell model was developed by MAEC stimulated with TNF-*α* and ox-LDL. Figure S3: the relative fluorescent intensity correlated logarithmically with the concentration of ICG-loaded Neu-balloon. Figure S4: fed with high cholesterol diet, the atherosclerotic plaques were progressed in the Apo E^−/−^ mice aorta. Figure S5: the acoustic intensity correlated with the extent of macrophage infiltration within the plaque and vulnerable index of the plaque. Figure S6: the fluorescent signal intensity increased along with the lipid-rich area in the aorta. Figure S7: biosafety of the Neu-balloons.

## References

[1] Soehnlein O. (2012). Multiple roles for neutrophils in atherosclerosis. *Circulation Research*.

[2] Asada Y., Yamashita A., Sato Y., Hatakeyama K. (2020). Pathophysiology of atherothrombosis: mechanisms of thrombus formation on disrupted atherosclerotic plaques. *Pathology International*.

[3] Libby P., Buring J. E., Badimon L. (2019). Atherosclerosis. *Nature Reviews Disease Primers*.

[4] Kaufmann B. A., Sanders J. M., Davis C. (2007). Molecular imaging of inflammation in atherosclerosis with targeted ultrasound detection of vascular cell adhesion molecule-1. *Circulation*.

[5] Xue J., Zhao Z., Zhang L. (2017). Neutrophil-mediated anticancer drug delivery for suppression of postoperative malignant glioma recurrence. *Nature Nanotechnology*.

[6] Kang T., Zhu Q., Wei D. (2017). Nanoparticles coated with neutrophil membranes can effectively treat cancer metastasis. *ACS Nano*.

[7] Drechsler M., Megens R. T., van Zandvoort M., Weber C., Soehnlein O. (2010). Hyperlipidemia-triggered neutrophilia promotes early atherosclerosis. *Circulation*.

[8] Su T., Feng X., Yang J. (2022). Polymer nanotherapeutics to correct autoimmunity. *Journal of Controlled Release*.

[9] Dancey J. T., Deubelbeiss K. A., Harker L. A., Finch C. A. (1976). Neutrophil kinetics in man. *The Journal of Clinical Investigation*.

[10] Nahrendorf M., Swirski F. K. (2015). Immunology. Neutrophil-macrophage communication in inflammation and atherosclerosis. *Science*.

[11] Marshall B. T., Long M., Piper J. W., Yago T., McEver R. P., Zhu C. (2003). Direct observation of catch bonds involving cell-adhesion molecules. *Nature*.

[12] Sundd P., Gutierrez E., Koltsova E. K. (2012). 'Slings' enable neutrophil rolling at high shear. *Nature*.

[13] Wu M., Zhang H., Tie C. (2018). MR imaging tracking of inflammation-activatable engineered neutrophils for targeted therapy of surgically treated glioma. *Nature Communications*.

[14] Ouyang J., Tang Z., Farokhzad N. (2020). Ultrasound mediated therapy: recent progress and challenges in nanoscience. *Nano Today*.

[15] Kaya M., Toma C., Wang J. (2012). Acoustic radiation force for vascular cell therapy: _in vitro_ validation. *Ultrasound in Medicine & Biology*.

[16] Yi L., Chen Y., Jin Q. (2020). Antagomir-155 attenuates acute cardiac rejection using ultrasound targeted microbubbles destruction. *Advanced Healthcare Materials*.

[17] Yuan H., Hu H., Sun J. (2018). Ultrasound microbubble delivery targeting intraplaque neovascularization inhibits atherosclerotic plaque in an APOE-deficient mouse model. *In Vivo*.

[18] Lindner J. R., Song J., Jayaweera A. R., Sklenar J., Kaul S. (2002). Microvascular rheology of definity microbubbles after intra-arterial and intravenous administration. *Journal of the American Society of Echocardiography*.

[19] Dlouha D., Prochazkova I., Eretova Z., Hubacek J. A., Parikova A., Pitha J. (2019). Influence of lipoprotein apheresis on circulating plasma levels of miRNAs in patients with high Lp(a). *Atherosclerosis Supplements*.

[20] Du X., Zhu M., Zhang T. (2022). The recombinant Eg.P29-mediated miR-126a-5p promotes the differentiation of mouse naive CD4^+^ T cells via DLK1-mediated notch1 signal pathway. *Frontiers in Immunology*.

[21] Li L., Ma W., Pan S. (2020). MiR-126a-5p limits the formation of abdominal aortic aneurysm in mice and decreases ADAMTS-4 expression. *Journal of Cellular and Molecular Medicine*.

[22] Chen W., Liu C., Ji X. (2021). Stanene-based nanosheets for *β*-elemene delivery and ultrasound-mediated combination cancer therapy. *Angewandte Chemie International Edition*.

[23] Zheng C., Li M., Ding J. (2021). Challenges and opportunities of nanomedicines in clinical translation. *BIO Integration*.

[24] Rotzius P., Thams S., Soehnlein O. (2010). Distinct infiltration of neutrophils in lesion shoulders in ApoE^−/−^ mice. *The American Journal of Pathology*.

[25] Chistiakov D. A., Bobryshev Y. V., Orekhov A. N. (2015). Neutrophil's weapons in atherosclerosis. *Experimental and Molecular Pathology*.

[26] Fenyo I. M., Gafencu A. V. (2013). The involvement of the monocytes/macrophages in chronic inflammation associated with atherosclerosis. *Immunobiology*.

[27] Feng X., Xu W., Li Z., Song W., Ding J., Chen X. (2019). Immunomodulatory nanosystems. *Science*.

[28] Tao W., Yurdagul A., Kong N. (2020). siRNA nanoparticles targeting CaMKII*γ* in lesional macrophages improve atherosclerotic plaque stability in mice. *Science Translational Medicine*.

[29] Yuan C., Li Y., Liu L. (2021). Experimental study on the compatibility and characteristics of a dual-target microbubble loaded with anti-miR-33. *International Journal of Nanomedicine*.

[30] Liu Y., Zhou Y., Xu J. (2021). Ultrasound molecular imaging-guided tumor gene therapy through dual-targeted cationic microbubbles. *Biomaterials Science*.

[31] Kong H., Ju E., Yi K. (2021). Advanced nanotheranostics of CRISPR/Cas for viral hepatitis and hepatocellular carcinoma. *Advanced Science*.

[32] Wallace C. S., Champion J. C., Truskey G. A. (2007). Adhesion and function of human endothelial cells co-cultured on smooth muscle cells. *Annals of Biomedical Engineering*.

[33] Yan F., Sun Y., Mao Y. (2018). Ultrasound molecular imaging of atherosclerosis for early diagnosis and therapeutic evaluation through leucocyte-like multiple targeted microbubbles. *Theranostics*.

