# Supplementary material for: Bionic Microbubble Neutrophil Composite for Inflammation-Responsive Atherosclerotic Vulnerable Plaque Pluripotent Intervention
Source: Research (Wash D C). 2022 Jun 3;2022:9830627. doi: 10.34133/2022/9830627 (PMC9188677; doi:10.34133/2022/9830627)
Supplement: Supplementary Materials — The supplementary materials are available by the publisher as online-only content, linked to the online manuscript. All the supplementary materials are summarized in a single file, including text, figures, and tables, as listed below. Figure S1: size distribution curve of the isolated neutrophils and prepared microbubbles. Figure S2: the inflammatory cell model was developed by MAEC stimulated with TNF-α and ox-LDL. Figure S3: the relative fluorescent intensity correlated logarithmically with the concentration of ICG-loaded Neu-balloon. Figure S4: fed with high cholesterol diet, the atherosclerotic plaques were progressed in the Apo E−/− mice aorta. Figure S5: the acoustic intensity correlated with the extent of macrophage infiltration within the plaque and vulnerable index of the plaque. Figure S6: the fluorescent signal intensity increased along with the lipid-rich area in the aorta. Figure S7: biosafety of the Neu-balloons. [file 9830627.f1.pdf]

Supporting information

Bionic Microbubble Neutrophil Composite for Inflammation-responsive Atherosclerotic Vulnerable Plaque Pluripotent Intervention

Fangfang Liu[1]†; Yang Mao[1]†; Jiaqi Yan[2]; Yu Sun[3]; Zhihua Xie[4]; Fei Li[3]; Fei Yan[3]*; Hongbo Zhang[2]*; Pengfei Zhang[1]*

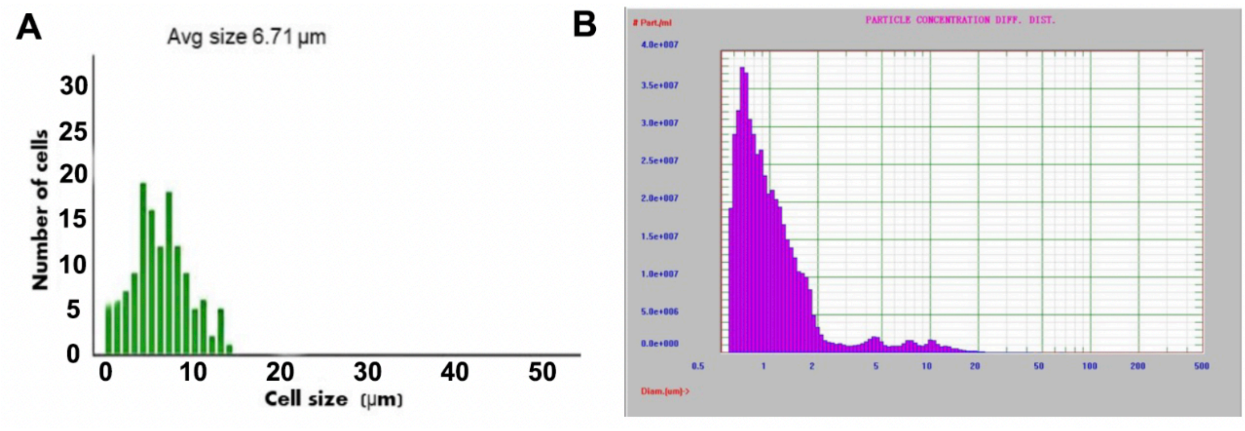

Figure S1 Size distribution curve of the isolated neutrophils and prepared microbubbles. (A) The average size of the isolated neutrophils from a typical batch was 6.71 μm. (B) The average size of the bare microbubbles from one batch was 1.43 μm.

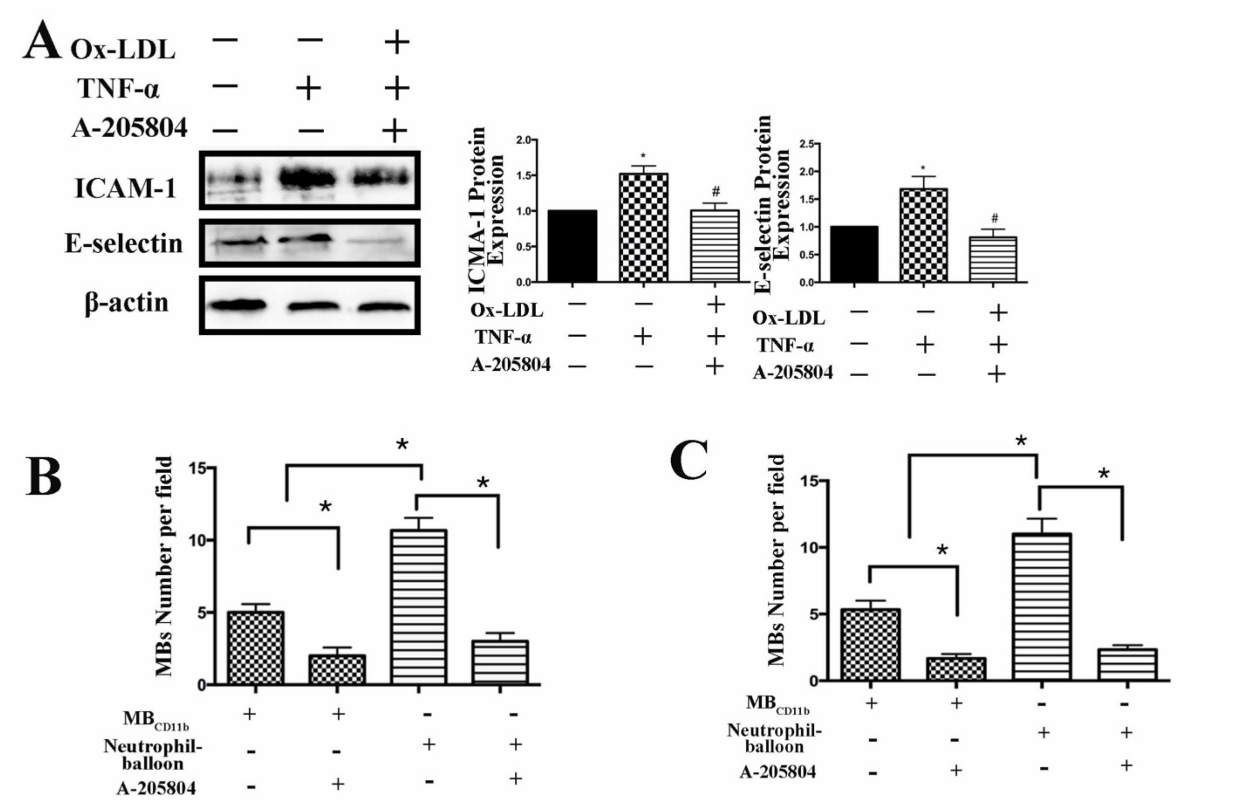

Figure S2 The inflammatory cell model was developed by MAEC stimulated with TNF-α and ox-LDL. (A) The expression of ICAM-1 and E-selectin, which should be abundant on the activated vascular endothelium membrane accounting for the inflammation responsive adhesion of neutrophils, had significantly increased. This over expression could be suppressed by 2,4-

disubstituted thieno [2,3-c] pyridine (A-205804). (B) Due to the inhibition of these inflammatory responding molecules, the adhesion of either MB_{CD11b} or Neu-balloons to the MAEC was hindered. However, in spite of the inhibition of ICAM-1 and E-selectin expression, the Neu-balloons maintained high affinity to the MAEC in the aid of neutrophil-parts of the Neu-balloons. (C) Similarly, under the high shear stress circumstance mimicked in parallel flow plate, strong adhesion of Neu-balloons to the MAEC was still notable even if the ICAM-1 and E-selectin were blocked by A-205804. * indicates P<0.01.

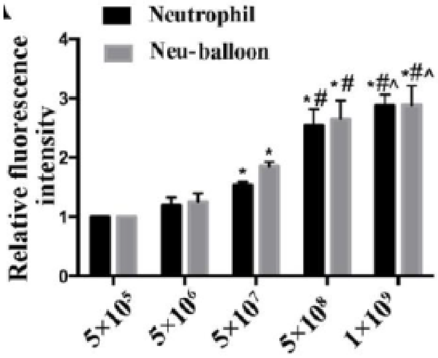

Figure S3 The relative fluorescent intensity correlated logarithmically with the concentration of ICG loaded Neu-balloon (R^2=0.97). * indicates $P < 0.05$ compared to 5×10^5/ml group, $^{\#}$ indicates $P < 0.05$ compared to 5×10^6/ml group, ^ indicates $P < 0.05$ compared to 5×10^7/ml group.

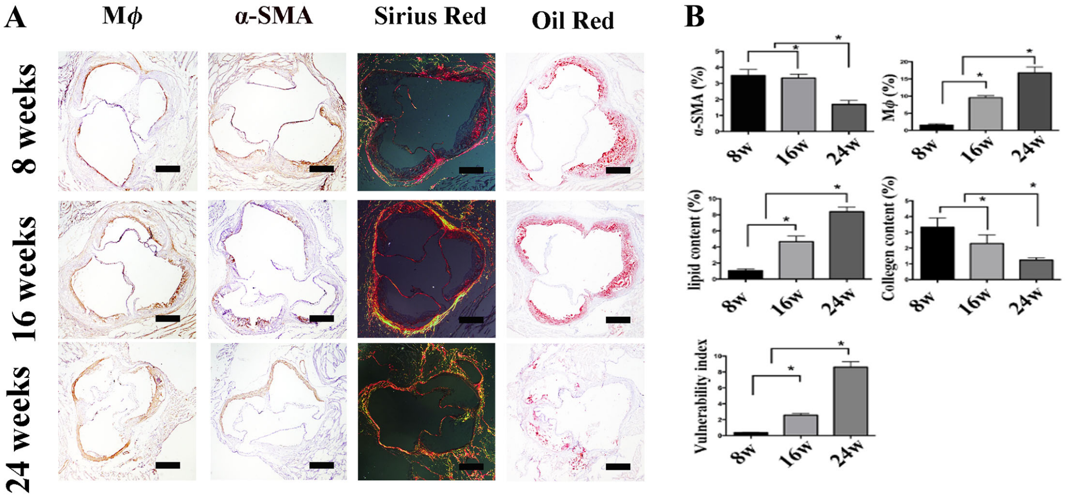

Figure S4 Fed with high cholesterol diet, the atherosclerotic plaques were progressed in the Apo E $^{-/-}$ mice aorta. (A) As revealed by histology and immunohistology, the contents of lipid (stained with Oil-Red) and macrophage (stained with anti-CD68) increased, while the contents of collagen (stained with Sirius Red) and smooth muscle cells (stained with anti-α-actin) decreased over time. (B) Statistical analysis of the histological images over time. The contents were expressed as the percentage of the positive staining area to the total plaque area. Obviously, the vulnerability of the plaques deteriorated in the mice fed till the end of 24 weeks, as the vulnerable index increased significantly (* indicates P<0.05 compared to Apo E -/- mice fed with atherogenic diets for 8 weeks).

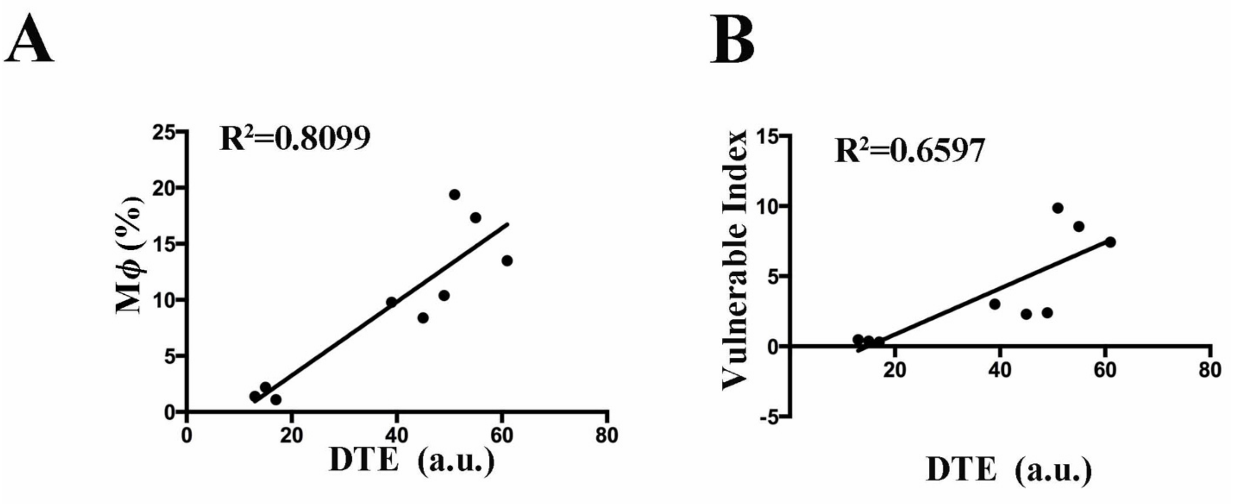

Figure S5 Statistical analysis between the acoustic intensity and the extent of macrophage infiltration within the plaque or vulnerable index of the plaque. (A) The acoustic intensity correlated very well and positively with the extent of macrophage infiltration within the plaque (R^2=0.8099, P<0.05). (B) The acoustic intensity correlated very well and positively with the vulnerable index of the plaque (R^2= 0.6597, P<0.05). DTE indicates differential targeted enhancement.

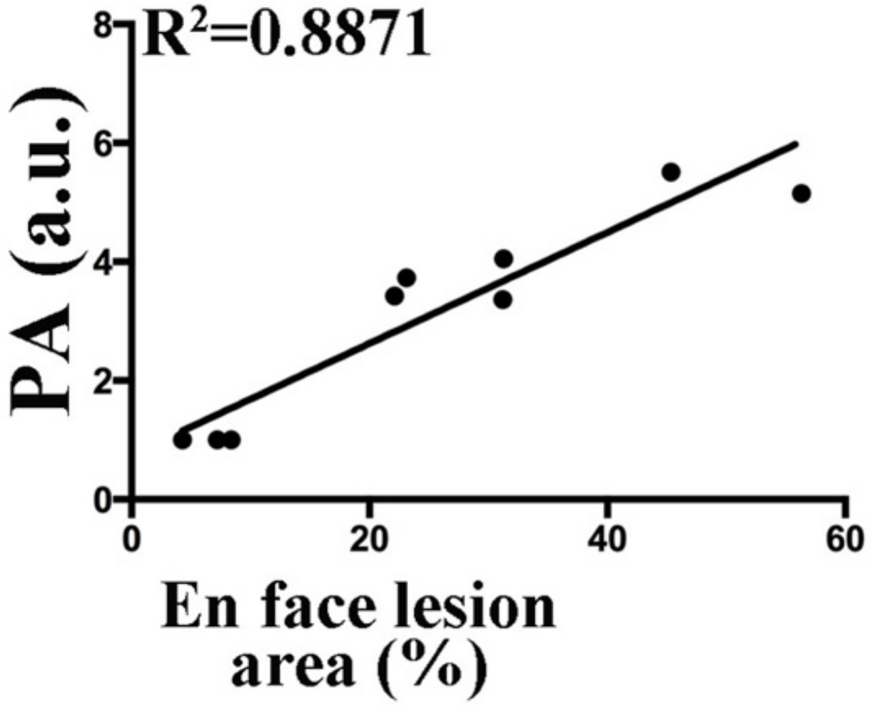

Figure S6 The fluorescent signal intensity increased along with the lipid-rich area in the aorta. The R-square of the correlation coefficient was 0.8871 (P<0.05) between the atherosclerotic lesion area and the fluorescent signal intensity acquired by photoacoustic microscopy.

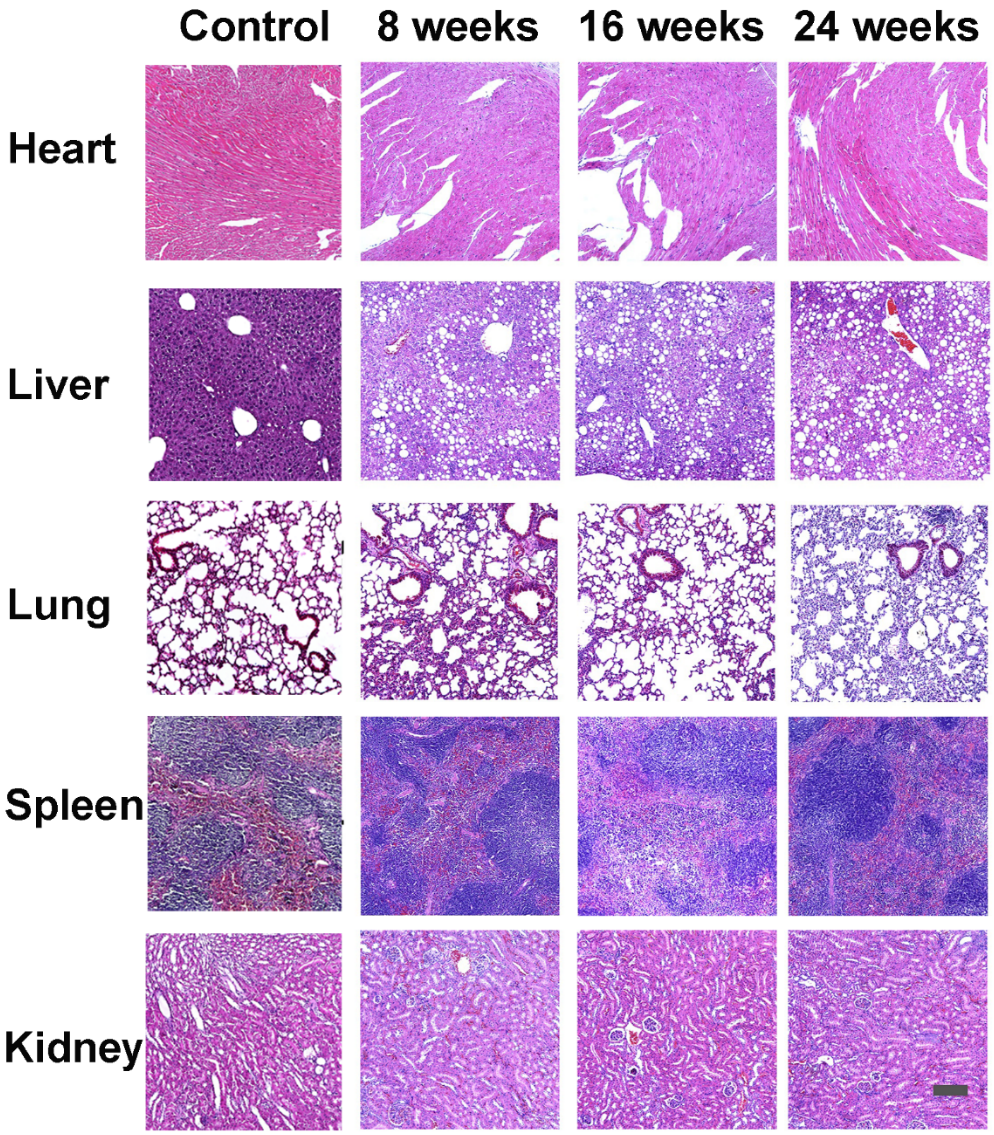

Figure S7 Biosafety of the Neu-balloons. In spite of different stage of the atherosclerotic mouse model, no damage could be noted in the heart, liver, lung, spleen or kidney tissues after the injection of Neu-balloons with concentration of 5×10^8 neutrophils/ml. Scale bar=50 μm.
